# Genetic and Serologic Properties of Zika Virus Associated with an Epidemic, Yap State, Micronesia, 2007 

**DOI:** 10.3201/eid1408.080287

**Published:** 2008-08

**Authors:** Robert S. Lanciotti, Olga L. Kosoy, Janeen J. Laven, Jason O. Velez, Amy J. Lambert, Alison J. Johnson, Stephanie M. Stanfield, Mark R. Duffy

**Affiliations:** * Centers for Disease Control and Prevention, Fort Collins, Colorado, USA

**Keywords:** Zika virus, laboratory diagnosis, RT-PCR, flavivirus, serology, research

## Abstract

Zika virus (ZIKV) is a mosquito-borne flavivirus first isolated in Uganda from a sentinel monkey in 1947. Mosquito and sentinel animal surveillance studies have demonstrated that ZIKV is endemic to Africa and Southeast Asia, yet reported human cases are rare, with <10 cases reported in the literature. In June 2007, an epidemic of fever and rash associated with ZIKV was detected in Yap State, Federated States of Micronesia. We report the genetic and serologic properties of the ZIKV associated with this epidemic.

## Abstract 2

One-sentence summary for table of contents: The full coding region nucleic acid sequence and serologic properties of the virus were identified.

Zika virus (ZIKV) is a mosquito-transmitted virus in the family *Flaviviridae* and genus *Flavivirus*. It was initially isolated in 1947 from blood of a febrile sentinel rhesus monkey during a yellow fever study in the Zika forest of Uganda (*1*). The virus was subsequently isolated from a pool of *Aedes africanus* mosquitoes collected in 1948 from the same region of the Zika forest; a serologic survey conducted at that time showed that 6.1% of the residents in nearby regions of Uganda had specific antibodies to ZIKV (*1*,*2*).

Over the next 20 years, several ZIKV isolates were obtained from *Aedes* spp. in Africa (*Ae*. *africanus*) and Malaysia (*Ae*. *aegypti*), implicating these species as likely epidemic or enzootic vectors (*3*–*5*). Several ZIKV human isolates were also obtained in the 1960s and 1970s from East and West Africa during routine arbovirus surveillance studies in the absence of epidemics (*6*–*8*). Additional serologic studies in the 1950s and 1960s detected ZIKV infections among humans in Egypt, Nigeria, Uganda, India, Malaysia, Indonesia, Pakistan, Thailand, North Vietnam, and the Philippines (*5*). These data strongly suggest widespread occurrence of ZIKV from Africa to Southeast Asia west and north of the Wallace line.

In 1977, ZIKV infection was confirmed among 7 patients in central Java, Indonesia, during an acute fever study (*9*). Data on these 7 ZIKV cases and several previously reported human infections indicated that clinical characteristics of infection with ZIKV included fever, headache, malaise, stomach ache, dizziness, anorexia, and maculopapular rash; in all cases infection appeared relatively mild, self-limiting, and nonlethal (*6*,*8*–*10*).

In April 2007, an epidemic of rash, conjunctivitis, and arthralgia was noted by physicians in Yap State, Federated States of Micronesia (*11*). Laboratory testing with a rapid assay suggested that a dengue virus (DENV) was the causative agent. In June 2007, samples were sent for confirmatory testing to the Arbovirus Diagnostic Laboratory at the Centers for Disease Control and Prevention (CDC, Fort Collins, CO, USA). Serologic testing by immunoglobulin (Ig) M–capture ELISA with DENV antigen confirmed recent flavivirus infection in several patients. Testing by reverse transcription–PCR (RT-PCR) with flavivirus consensus primers generated DNA fragments, which when subjected to nucleic acid sequencing, demonstrated ≈90% nucleotide identity with ZIKV. These findings indicated that ZIKV was the causative agent of the Yap epidemic. We report serologic parameters of the immune response among ZIKV-infected humans, data on estimated levels of viremia, and the complete coding region nucleic acid sequence of ZIKV associated with this epidemic.

## Methods

### Analysis of Patient Samples

Details of the epidemic, including clinical and laboratory findings for all patients, will be reported elsewhere (M.R. Duffy et al., unpub. data). A subset of ZIKV-infected patients for whom acute- and convalescent-phase paired serum specimens had been collected was analyzed by using several serologic assays to evaluate the extent of cross-reactivity to several related flaviviruses. Patients were classified as primary flavivirus/ZIKV infected or secondary flavivirus/ZIKV probable infected. Primary flavivirus/ZIKV–infected patients were those in whom acute-phase serum specimens (<10 days) had no detectible antibodies (by IgG ELISA and plaque-reduction neutralization test [PRNT]) to any of the heterologous flaviviruses tested (Tables 1, 2) and were either IgM-positive in their acute-phase specimen or IgM and IgG positive for ZIKV in a convalescent-phase specimen (seroconversion). Secondary flavivirus/ZIKV probable–infected patients were those who had detectable antibodies to >1 heterologous flaviviruses in their acute-phase specimen and were also IgM positive for ZIKV in their acute-phase specimen, or IgM and IgG positive for ZIKV in their convalescent-phase specimen. The designation “ZIKV probable” was used because secondary flavivirus infections demonstrate extensive cross-reactivity with other flaviviruses, and in some cases, higher serologic reactivity to the original infecting flavivirus (“original antigenic sin” phenomenon). Thus, in secondary flavivirus infections shown in Tables 1 and 2, serologic data alone is insufficient to confirm ZIKV as the recently infecting flavivirus. However, these secondary flavivirus/ZIKV probable infections were likely recent ZIKV infections because ZIKV was the only virus detected during the epidemic in Yap, a relatively small and isolated island (*11*).

**Table 1:** IgG and IgM testing with heterologous flaviviruses of patients infected with ZIKV, Yap State, Micronesia, 2007*

Patient	Days after onset	IgG		IgM					
		ZIKV		ZIKV	DENV	YFV	JEV	MVEV	WNV
Primary flavivirus ZIKV									
822a	5	1.5		23.2	1.3	1.4	1.7	1.1	–
822b	10	1.2		39.5	1.2	1.0	2.4	1.2	–
822c	24	3.3		13.1	2.7	0.63	1.8	1.3	–
830a	2	1.1		1.3	4.4	0.48	4.4	2.9	–
830b	21	1.8		16.3	1.9	0.63	1.3	1.6	–
849a	3	1.5		4.5	0.92	0.95	1.2	0.66	–
849b	18	3.0		18.2	2.2	1.0	2.7	1.5	–
862a	6	1.9		25.4	1.7	1.1	1.8	1.0	–
862b	20	2.6		15.4	2	1.1	2.3	1.1	Eq
Secondary flavivirus ZIKV (probable)									
817a	1	5.9		1.4	1.7	0.8	1.7	0.7	–
817b	19	5.7		8.1	5.1	2.1	1.7	1.0	–
833a	1	3.4		1.7	3.7	1.0	2.8	1.3	–
833b	19	8.2		3.1	2.3	0.9	2.5	1.3	–
844a	2	3.8		3.8	6.8	2.0	21.5	0.7	–
844b	16	8.5		12.7	14.9	7.0	42.9	1.6	–
955a	1	5.0		1.8	3.7	1.0	3.4	2.4	Eq
955b	14	26.6		10.9	3.4	0.8	1.7	4.0	Eq
968a	1	4.0		1.7	1.3	0.6	1.2	1.2	–
968b	3	12.3		20.4	2.9	0.8	0.9	2.0	–
839a	3	1		0.92	3.4	0.7	2.7	2.1	–
839b	20	4.9		17.2	2.2	2.1	1.9	1.8	–
847a	5	0.9		0.94	4.1	4.1	2.3	1.3	–
847b	8	14.1		21.5	1.4	3.3	1.1	2.6	–

*Ig, immunoglobulin; ZIKV, Zika virus; DENV, dengue virus type 1–4 mixture; YFV, yellow fever virus; JEV, Japanese encephalitis virus; MVEV, Murray Valley encephalitis virus; WNV, West Nile virus; –, negative. Eq, result in equivocal range of the assay. IgG and IgM testing was conducted by ELISA except for WNV, which was tested by microsphere assay; ELISA values are patient optical densities divided by negative control optical densities; <2, negative; 2–3 equivocal; >3 positive.

**Table 2:** Neutralization testing with heterologous flaviviruses of patients infected with ZIKV, Yap State, Micronesia, 2007*

Patient	Days after onset	PRNT_90_ titer									
		ZIKV	DENV1	DENV2	DENV3	DENV4	JEV	YFV	WNV	SLEV	MVEV
Primary flavivirus ZIKV											
822a	5	320	<10	<10	<10	<10	<10	<10	<10	<10	<10
822b	10	2,560	10	10	10	10	<10	<10	<10	<10	<10
822c	24	5,120	10	10	10	10	<10	<10	<10	<10	<10
830a	2	<10	<10	NT‡	NT	NT	NT	NT	NT	NT	NT
830b	21	2,560	<10	<10	<10	<10	<10	<10	<10	<10	<10
849a	3	<10	<10	<10	<10	<10	<10	<10	<10	<10	<10
849b	18	10,240	<10	<10	<10	<10	<10	20	<10	<10	<10
862a	6	320	<10	<10	<10	<10	<10	<10	<10	<10	<10
862b	20	2,560	10	10	<10	<10	<10	<10	<10	10	<10
Secondary flavivirus ZIKV (probable)											
817a	1	80	80	160	320	160	<10	<10	<10	40	40
817b	19	10,240	2,560	20,480	5,120	5,120	20	320	160	1,280	640
833a	1	160	320	80	40	20	<10	<10	<10	<10	<10
833b	19	81,920	20,480	5,120	5,120	1,280	<10	<10	80	320	320
844a	2	20	1,280	640	320	160	<10	<10	5	20	20
844b	16	10,240	40,980	10,240	5,120	1,280	5	<10	160	640	640
955a	1	40	1,280	640	160	320	<10	<10	<10	20	20
955b	14	163,840	81,920	20,480	10,240	5,120	10	<10	640	2,560	1,280
968a	1	80	320	320	80	40	<10	<10	<10	40	20
968b	3	10,240	640	640	160	160	<10	<10	10	40	20
839a	3	<10	<10	10	<10	<10	<10	40	<10	<10	<10
839b	20	10,240	40	320	80	80	<10	640	40	80	80
847a	5	<10	<10	<10	<10	<10	<10	640	<10	<10	<10
847b	8	2,560	40	320	160	40	<10	1,280	80	320	320

*PRNT_90_ titer, 90% plaque-reduction neutralization test titer; ZIKV, Zika virus; DENV, dengue virus; JEV, Japanese encephalitis virus; YFV, yellow fever virus; WNV, West Nile virus; SLEV, St. Louis encephalitis virus; MVEV, Murray Valley encephalitis virus; NT, not tested (sample depleted).

### Serologic Testing

Acute- and convalescent-phase serum samples were tested by IgG ELISA with ZIKV antigen as described for detection of IgG to arboviruses (*12*). Samples were also tested by IgM ELISA as described with the following viral antigens: ZIKV, DENV 1–4 mixture, yellow fever virus (YFV), Japanese encephalitis virus, and Murray Valley encephalitis virus (*13*). Testing for IgM to West Nile virus (WNV) and St. Louis encephalitis virus was performed by using a microsphere immunoassay (*14*). Ratios of patient optical density values to negative control values (P/Ns) were calculated for IgG and IgM ELISAs. Values >3 were considered positive, and values 2–3 were considered equivocal. Neutralizing antibody titers were determined by using a PRNT with a 90% cut-off value (*15*).

### Real-Time RT-PCR

Two real-time primer/probe sets specific for the ZIKV 2007 strain were designed by using ZIKV 2007 nucleotide sequence data in the PrimerExpress software package (Applied Biosystems, Foster City, CA, USA). Primers were synthesized by Operon Biotechnologies (Huntsville, AL, USA) with 5-FAM as the reporter dye for the probe (Table 3). All real-time assays were performed by using the QuantiTect Probe RT-PCR Kit (QIAGEN, Valencia, CA, USA) with amplification in the iCycler instrument (Bio-Rad, Hercules, CA, USA) following the manufacturer’s protocol. Specificity of the ZIKV primers was evaluated by testing the following viral RNAs, all of which yielded negative results: DENV-1, DENV-2, DENV-3, DENV-4, WNV, St. Louis encephalitis virus, YFV, Powassan virus, Semliki Forest virus, o’nyong-nyong virus, chikungunya virus, and Spondweni virus (SPOV).

**Table 3:** Description and performance characteristics of Zika virus real-time RT-PCR primer/probe sets*

Primer	Genome position†	Sequence (5′ → 3′)	Sensitivity, no. copies	Specificity‡
ZIKV 835	835–857	TTGGTCATGATACTGCTGATTGC		
ZIKV 911c	911–890	CCTTCCACAAAGTCCCTATTGC	100	ZIKV
ZIKV 860-FAM	860–886	CGGCATACAGCATCAGGTGCATAGGAG		
ZIKV 1086	1086–1102	CCGCTGCCCAACACAAG		
ZIKV 1162c	1162–1139	CCACTAACGTTCTTTTGCAGACAT	25	ZIKV
ZIKV 1107-FAM	1107–1137	AGCCTACCTTGACAAGCAGTCAGACACTCAA		

*RT-PCR, reverse transcription–PCR; ZIKV, Zika virus. †Based on ZIKV MR 766 GenBank accession no. AY632535. ‡ZIKV specificity indicates a positive result with ZIKV only and no reactivity with dengue virus-1 (DENV-1), DENV-2, DENV-3, DENV-4, West Nile virus, St. Louis encephalitis virus, yellow fever virus, Powassan virus, Semliki Forest virus, o’nyong-nyong virus, chikungunya virus, and Spondweni virus.

Sensitivity of the ZIKV real-time assay was evaluated by testing dilutions of known copy numbers of an RNA transcript copy of the ZIKV 2007 sequence. Copy numbers of RNA were determined by using the Ribogreen RNA-specific Quantitiation Kit (Invitrogen) and the TBE-380 mini-fluorometer (Turner Biosystems, Sunnyvale, CA, USA). RNA transcripts ranging from 16,000 to 0.2 copies were tested in quadruplicate to determine the sensitivity limit and to construct a standard curve for estimating the genome copy number of ZIKV in patient samples. All serum samples obtained during the epidemic were tested for ZIKV RNA by using this newly designed real-time RT-PCR. Concentration of viral RNA (copies/milliliter) was estimated in ZIKV-positive patients by using the standard curve calculated by the iCycler instrument (Table 4). All RT-PCR–positive specimens were placed on monolayers of Vero, LLC-MK2, and C6/36 cells to isolate virus; no specimens showed virus replication.

**Table 4:** Results of quantitative real-time RT-PCR of samples from ZIKV-positive patients, Yap State, Micronesia, 2007*

Patient	Days after onset	ZIKV real-time RT-PCR			
		Ct-860†	Ct-1107†	Result	Estimated copies/mL‡
824	1	34.3	34.7	+	11,647
939	2	32.0	32.4	+	67,817
947	2	34.3	33.9	+	21,495
949	2	35.1	35.1	+	8,573
969	1	29.4	29.3	+	728,800
037	1	32.1	32.5	+	62,816
830a	2	30.7	30.0	+	426,325
847a	5	34.8	34.7	+	11,647
950a	0	32.2	32.7	+	53,894
943	3	37.6	35.6	+	5,845
952	1	29.3	29.5	+	625,280
958	11	29.9	30.3	+	338,797
970	1	35.5	34.8	+	10,788
42	0	32.9	33.6	+	27,048
941	3	31.1	38.0	+	930
964	0	38.3	37.6	+	1,263
063a	2	37.5	38.0	+	930

*RT-PCR, reverse transcription­–PCR; ZIKV, Zika virus; Ct, crossing threshold; +, positive. †Ct values with primer set 835/911c/860-FAM or 1086/1162c/1107-FAM. Values <38.5 are positive. ‡Estimated by testing quantitated dilutions of ZIKV RNA transcripts and standard curve calculation generated by the iCycler instrument (Bio-Rad, Hercules, CA, USA; see Methods).

### Nucleic Acid Sequencing and Phylogenetic Analysis

RNA was extracted from patient samples that demonstrated the highest concentration of ZIKV RNA determined by the real-time assay, and for which sufficient sample volume was available (patients 824, 037, 830a, and 958). Briefly, RNA was extracted from 150 μL of serum by using the QIAamp Viral RNA Mini Kit (QIAGEN), and RNA was eluted with 75 μL of RNase-free water. A series of RT-PCRs was performed with each RNA preparation by using primer pairs designed to generate overlapping DNA fragments that spanned the entire polyprotein coding region of the virus. Primers were designed by using the ZIKV MR 766 prototype virus coding region sequence (GenBank accession no. AY632535) and the PrimerSelect software module of the LaserGene package (DNASTAR Inc., Madison, WI, USA). Several primers initially failed to amplify because of sequence mismatches between ZIKV MR 766 and ZIKV Yap 2007. Therefore, primers were redesigned by using newly generated DNA sequence data, and a “genome walking” approach was used to derive complete coding region sequence data. The complete list of amplification and sequencing primers is available upon request.

All RT-PCRs were performed with 10 μL of RNA by using the OneStep RT-PCR Kit (QIAGEN) following the manufacturer’s protocol. DNAs were analyzed by 2% agarose gel electrophoresis, and bands of the predicted size were excised from the gel and purified by using the QIAquick Gel Extraction Kit (QIAGEN). Purified DNAs were subjected to nucleic acid sequence analysis with sequencing primers spaced ≈500 bases apart on both strands of the DNA fragments by using the ABI BigDye Terminator V3.1 Ready Reaction Cycle Sequencing Mixture (Applied Biosystems). Nucleotide sequence was determined by capillary electrophoresis by using the ABI 3130 genetic analyzer (Applied Biosystems) following the manufacturer’s protcol. Raw sequence data were aligned and edited by using the SeqMan module of LaserGene (DNASTAR Inc.). Because of insufficient sample volume, no patient RNA was sufficient to generate DNA that included the entire coding region. Therefore, DNA data obtained from 4 patients was combined to generate a consensus sequence heretofore designated the ZIKV 2007 epidemic consensus (EC) sequence (GenBank accession no. EU545988).

**Figure 1:**
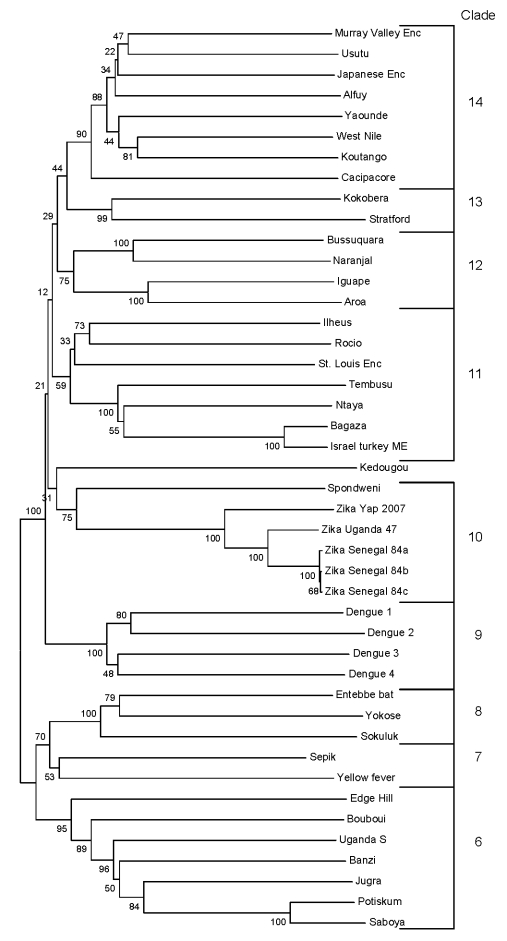
Phylogenetic tree constructed from nucleic acid data from flavivirus nonstructural protein 5 region by the neighbor-joining algorithm in MEGA (www.megasoftware.net). Numbers to the left of the nodes are bootstrap percentages (2,000 replications) for clades. Clade numbers correspond to clades identified by Kuno et al. (*16*). Enc, encephalitis; ME, meningoencephalitis.

The complete coding region of ZIKV 2007 EC or the nonstructural protein 5 (NS5) gene subregion was aligned with all available flavivirus sequences in GenBank by using the Clustal W algorithm within the MEGA version 4 software package (www.megasoftware.net). Phylogenetic trees were constructed by using either the complete coding region or the NS5 region because a large number of NS5 sequences were available in GenBank and trees for the NS5 region have been constructed (*16*). Additional ZIKV strains from the CDC/World Health Organization reference collection (strains 41662, 41524, and 41525) isolated from *Aedes* spp. mosquitoes collected in Senegal in 1984 were also amplified by RT-PCR in the NS5 region and subjected to nucleic acid sequencing as described above and included in the NS5 region analysis. Trees were constructed from coding region data or from NS5 data by MEGA 4 from aligned nucleotide sequences. We used maximum parsimony, neighbor-joining, or minimum evolution algorithms with 2,000 replicates for bootstrap support of tree groupings. All trees generated nearly identical topology; only the neighbor-joining NS5 tree is shown (Figure 1).

## Results

### Serologic Analysis

Tables 1 and 2 show results of analysis for IgG and IgM and PRNTs of all acute- and convalescent-phase paired specimens obtained during the epidemic. Specimens were divided into primary and secondary infections on the basis of antibody testing results of acute-phase specimens. IgM antibody response in primary flavivirus/ZIKV–infected patients was specific for ZIKV. However, all of these patients showed some limited degree of cross-reactivity with heterologous flaviviruses. Patient 830a showed IgM-positive results with DENV and Japanese encephalitis virus, whereas all patients showed equivocal results (P/N 2–3) with several of the flaviviruses tested, suggesting low levels of cross-reactivity. PRNT_90_ results also showed that the neutralizing antibody response among primary flavivirus/ZIKV–infected patients was highly specific. Most convalescent-phase PRNT titers for heterologous flaviviruses were negative and rarely exceeded 10 (20 in 1 instance; patient 849b).

Most patient specimens from the Yap epidemic tested were secondary flavirius infections as determined by criteria described for antibody to flavivirus in acute-phase specimens. A subset of these patients for whom acute- and convalescent-phase specimens were available was tested for reactivity against heterologous flaviviruses; results are shown in Tables 1 and 2. In contrast to primary flavivirus/ZIKV–infected patients, secondary flavivirus–infected patients showed a high degree of serologic cross-reactivity with other flaviviruses. Six of 7 patients were positive for IgM against >1 of the heterologous flaviviruses tested, and all demonstrated low levels of cross-reactive IgM as shown by a P/N value in the equivocal range. PRNT_90_ results showed that among secondary flavivirus/ZIKV–probable patients, the neutralizing antibody response was higher to ZIKV and more cross-reactive, a finding commonly observed among secondary flavivirus infections. A >4-fold PRNT_90_ titer between ZIKV and heterologous flaviviruses was observed in only 3 of the 7 patients. In all other cases, the PRNT difference between ZIKV and other flaviviruses tested was <2-fold; in 2 patients (817b and 844b) the PRNT titer was higher for 1 of the heterologous flaviviruses. The PRNT result for the acute-phase specimen from patient 847 suggests previous vaccination with YFV. The convalescent-phase specimen from patient 847 showed a high titer to YFV, a demonstration of the previously described “original antigenic sin” phenomenon observed among flaviviruses (*17*).

### Real-Time RT-PCR

A real-time RT-PCR was developed by using newly derived sequence data obtained from several ZIKV-infected patients. All acute-phase specimens obtained during the Yap epidemic (n = 157) were tested in this assay with 2 unique primer/probe sets. Seventeen samples were positive, 10 were equivocal, and 130 were negative (data not shown). The equivocal designation indicates that a particular sample was positive by only 1 of the 2 primer sets or showed crossing thresholds >38.5, which suggests either a false-positive result or a sample with low levels of ZIKV RNA below the defined cut-off of the assay. Table 4 shows estimated viral concentrations of the 17 ZIKV-positive specimens. The viral RNA concentrations were ≈900–729,000 copies/mL. Most (15 of 17) of the ZIKV-positive samples were from specimens collected <3 days after onset; however, 1 specimen (patient 958) collected on day 11 after onset was positive with an estimated titer of ≈339,000 copies/mL.

### Nucleic Acid Sequence and Phylogenetic Analysis

Several RT-PCR–positive serum specimens were selected, and RNA was amplified by RT-PCR to generate DNA sequence data for the complete coding region. Because of limited specimen volume, the complete coding region genome sequence was only obtainable by combining sequence data from DNA fragments generated from 4 patients. Thus, the designation EC sequence is used to indicate that the sequence was derived from multiple patients during the epidemic. The exact contribution of sequence data from each patient is available upon request. However, the following points should be noted. Approximately 96% of the complete coding region was obtained from 3 patients; sequence data from the fourth patient was used primarily to fill in short gaps in the data. Second, ≈50% of the coding region data was derived from a complete overlap of data from >2 patients; in these overlap regions the sequence identity between different patients was ≈100%. Only 2-nt differences between patients were noted within the overlapping regions, strongly suggesting that 1 ZIKV strain circulated during the epidemic.

Percentage identity over the entire coding region of ZIKV 2007 EC sequence, when compared with the prototype ZIKV (MR 766, isolated in 1947), was 88.9% and 96.5% at the nucleotide and amino acid levels, respectively. Phylogenetic trees constructed from the complete coding region of all available flaviviruses generated by a variety of methods (neighbor-joining, maximum-parsimony, or minimum-evolution) showed the same overall topology, with the ZIKV prototype and 2007 EC virus placed in a unique clade (clade 10) within the mosquito-borne flavivirus cluster previously described by Kuno et al. (*16*). Alignment with phylogenetic tree construction by neighbor-joining, maximum-parsimony, or minimum-evolution algorithms was also performed for the NS5 region of all available flaviviruses because extensive sequencing and phylogenetic analysis have been conducted for this region (*16*).

Three additional ZIKV strains isolated from Senegal in 1984 and sequenced in this study were also included in a tree. This NS5 tree demonstrated similar topology to the complete coding region tree, with all ZIKVs placed within a unique clade (clade 10) along with SPOV. Figure 1 shows the NS5 tree with only mosquito-borne flaviviruses (cluster) displayed. This NS5 tree also shows that within the Zika/Spondweni clade there appear to be 3 branches among ZIKVs: Nigerian ZIKVs, prototype MR766, and 2007 Yap virus. Percentage identity among these ZIKVs confirms the tree topology, in which ZIKV 2007 EC is most distally related to East and West African ZIKV strains (data not shown).

**Figure 2:**
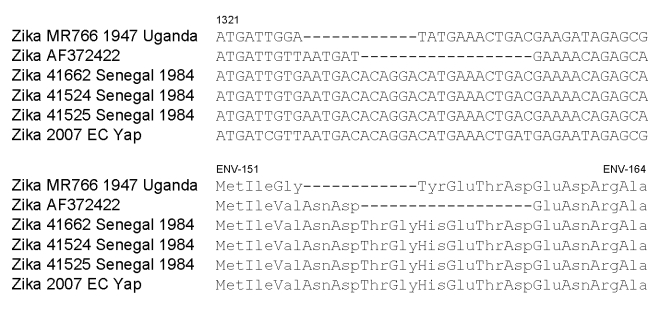
Alignment of nucleotide and amino acid sequences adjacent to the envelope (ENV)–154 glycosylation site of Zika virus strains. Dashes indicate deletions. EC, epidemic consensus.

The predicted amino acid sequence of ZIKV 2007 EC contains the Asn-X-Ser/Thr glycosylation motif at position 154 in the envelope glycoprotein, found in many flaviviruses, yet absent by deletion in the prototype ZIKV MR 766. This region of the prototype virus, along with 3 ZIKVs isolated from Senegal in 1984, was sequenced (Figure 2). Included in this alignment is a ZIKV isolate from GenBank (accession no. AF372422). Sequencing confirmed that prototype ZIKV MR766 has a 4-aa (12-nt) deletion when compared with ZIKV 2007 EC virus and ZIKVs from Senegal.

## Discussion

Historically, ZIKV has rarely been associated with human disease, with only 1 small cluster of human cases in Indonesia reported (*9*). We report a widespread epidemic of human disease associated with ZIKV in Yap State in 2007. ZIKV epidemics may have occurred but been misdiagnosed as dengue because of similar clinical symptoms and serologic cross-reactivity with DENVs. Our serologic data indicate that ZIKV-infected patients can be positive in an IgM assay for DENVs, particularly if ZIKV is a secondary flavivirus infection. If ZIKV is the first flavivirus encountered, our data indicate that cross-reactivity is minimal. However, when ZIKV infection occurs after a flavivirus infection, our data indicate that the extent of cross-reactivity in the IgM assay is greater. Therefore, if ZIKV infections occur in a population with DENV (or other flavivirus) background immunity, our data suggest that extensive cross-reactivity in the dengue IgM assay will occur, which could lead to the erroneous conclusion that dengue caused the epidemic. Whether this cross-reactivity has occurred is open to speculation. However, reexamination of specimens from dengue epidemics may provide an answer. In addition, use of virus isolation or RT-PCR for laboratory diagnosis of dengue infections would also prevent this misinterpretation. Therefore, use of virus detection assays in dengue epidemics should be a component of laboratory testing algorithms.

Levels of viremia among ZIKV-infected patients were relatively low. Unfortunately, measurement of concentration of infectious ZIKV was not possible because a virus isolate was not obtained from any patient during the epidemic. Absence of a ZIKV 2007 isolate also precluded use of a ZIKV 2007 isolate to generate a standard curve in the RT-PCR, which in turn could have estimated the concentration of infectious virus within patients. An estimation of the number of genome copies circulating in ZIKV-infected patients was calculated by using an RNA transcript and provides some indication of infectious virus concentration in ZIKV-infected patients. If one assumes a ratio range of 200–500 genome copies per infectious virus particle, a range reported for several flaviviruses, then the copies/milliliter values in Table 4 would be in the range of ≈2–3,500 infectious virus particles/mL, with only 4 specimens in which ZIKV exceeded 1,000 infectious units/mL (*18*,*19*). These findings may partially explain why ZIKV was not isolated, especially if one considers that shipping samples to our laboratory took ≈1 week, and shipping conditions were not conducive to virus isolation. These concentration estimates are also consistent with those of a study in which a ZIKV-infected human volunteer showed low viremia; virus was isolated only on day 4, and the volunteer was unable to infect *Ae*. *aegypti* mosquitoes that fed on the patient during the acute stage of disease (*10*).

Although generation of a complete coding region nucleic acid sequence by using a combination of patient samples from the epidemic is an unconventional approach, it was performed out of necessity because of limited volumes of patient samples. However, the extent of agreement among overlapping regions confirms that the sequence obtained accurately represents the virus associated with the epidemic. Nucleic acid sequence of ZIKV 2007 showed divergence (11%) from the prototype strain (MR766) isolated in 1947. However, the predicted amino acid sequence is fairly conserved (96%), which is likely the result of the selective pressure maintained on the virus because replication occurs in vertebrate hosts and arthropod vectors.

Phylogenetic trees based on the complete coding region or the NS5 region confirm results of a study in which ZIKV was classified in a unique clade among the mosquito-borne flaviviruses and most closely related to SPOV (*16*). The NS5 mosquito-borne flavivirus tree (Figure 1), which includes additional ZIKV isolates, confirms these relationships and suggests that there are 3 subclades among ZIKV isolates that reflect geographic origin. Senegal ZIKVs and prototype virus from Uganda may represent West and East African lineages, respectively. The 2007 ZIKV is distantly related to these 2 African subclades and may represent divergence from a common ancestor with spread throughout Southeast Asia and the Pacific. Human ZIKV cases were detected in peninsular Malaysia in 1980, which confirms that ZIKV was active in this region before 2007 (*9*). Additional sequence analysis of other temporally and geographically distinct ZIKV strains is needed to further elucidate relationships among these viruses.

Of particular interest is an additional 12 nt in the envelope gene (corresponding to 4 aa) in our ZIKV isolate that were not present in the ZIKV prototype virus (Figure 2). This difference is noteworthy because these 4 aa correspond to the envelope protein 154 glycosylation motif found in many flaviviruses and associated in some instances with virulence. This glycosylation motif is also absent because of a 6-aa deletion in the ZIKV isolate obtained from GenBank (accession no. AF372422); however, the geographic and temporal origins of this virus were not available. Loss of the envelope protein 154 glycoslyation site has been observed in some flaviviruses, and in the case of Kunjin virus has been shown to occur during passage. However, with Kunjin virus, the glycosylation site motif was lost because of a 1-base mutation, rather than a deletion, that altered the N-X-S/T sequon (*20*). Loss of this glycosylation site by a 4-aa deletion has also been observed in several lineage-2 WNV strains when compared with all other WNV strains (*21*).

The glycoslyation motif in WNV may be lost during extensive mouse brain passage; however, no direct evidence exists to support this hypothesis (*21*). This process may occur in ZIKV; the glycoslyation motif in MR 766 may have been present in earlier passages of prototype MR766 and lost during extensive mouse brain passage. However, earlier passage strains of MR766 were not available for investigating this hypothesis. Alternatively, the presence or absence of this glycosylation motif may represent an ancient evolutionary event with subsequent divergence of 2 ZIKV types with or without the E-154 glycosylation site amino acids. Sequence data derived from 3 additional ZIKV isolates from Senegal showed that glycosylation is intact in these isolates, which suggests evolutionary divergence. More extensive sequence analysis of available ZIKV strains of various temporal, geographic, and passage histories may provide some insight into this issue.
